# Radiotherapy in patients with distant metastatic breast cancer

**DOI:** 10.1186/1748-717X-9-126

**Published:** 2014-05-30

**Authors:** Kirsten Steinauer, Markus Wolfram Gross, Dorothy Jane Huang, Serenella Eppenberger-Castori, Uwe Güth

**Affiliations:** 1 Department of Radiation Therapy and Radiation Oncology, Cantonal Hospital Winterthur, Brauerstrasse 15, Winterthur CH-8401, Switzerland; 2 Breast Center “SenoSuisse”, Brauerstrasse 15, Winterthur CH-8401, Switzerland; 3 Department of Radiation Therapy and Radiation Oncology, University Hospital Basel (UHB), Petersgraben 4, Basel CH-4031, Switzerland; 4 UHB, Department of Gynecology and Obstetrics, Spitalstrasse 21, Basel CH-4031, Switzerland; 5 UHB, Institute of Pathology, Schönbeinstrasse 40, Basel CH-4031, Switzerland; 6 Department of Gynecology & Obstetrics, Cantonal Hospital Winterthur, Brauerstrasse 15, Winterthur CH-8401, Switzerland

**Keywords:** Breast cancer, Distant metastases, Palliative radiotherapy

## Abstract

**Background:**

The study evaluates frequency of and indications for disease-related radiotherapy in the palliative breast cancer (BC) situation and analyzes in which phase of the palliative disease course radiotherapy was applied.

**Patients & methods:**

340 patients who developed distant metastatic disease (DMD) and died (i.e. patients with completed disease courses) were analyzed.

**Results:**

165 patients (48.5%) received palliative radiotherapy (255 series, 337 planning target volumes) as a part of palliative care. The most common sites for radiotherapy were the bone (217 volumes, 64.4% of all radiated volumes) and the brain (57 volumes, 16.9%). 127 series (49.8%) were performed in the first third of the metastatic disease survival (MDS) period; 84 series (32.8%) were performed in the last third. The median survival after radiotherapy was 10 months. Patients who had received radiation were younger compared to those who had no radiation (61 vs. 68 years, p < 0.001) and had an improved MDS (26 vs. 14 months, p < 0.001). Compared to rapidly progressive disease courses with short survival times, in cases where effective systemic therapy achieved a longer MDS (≥24 months), radiotherapy was significantly more often a part of the multimodal palliative therapy (52.1% vs. 37.1%, p = 0.006).

**Conclusions:**

In a cohort of BC patients with DMD, nearly one half of the patients received radiotherapy during the palliative disease course. In a palliative therapy approach, which increasingly allows for treatment according to the principles of a chronic disease, radiotherapy has a clearly established role in the therapy concept.

## Introduction

In Western countries, approximately 5-10% of all breast cancer (BC) patients present with distant metastases at initial diagnosis (primary metastatic disease). Depending on prognostic factors, up to 30% of node-negative and up to 70% of node-positive BC patients develop distant metastases during the course of their illness (secondary metastatic disease) [1]. The prognoses and clinical courses of patients with distant metastatic BC vary considerably depending on host and tumor characteristics. Once distant metastases occur, BC remains a treatable condition but is no longer considered curable [1-3]. In this situation, radiotherapy might be performed with palliative intention and the primary goals of treatment include prevention and palliation of symptoms, maintenance or improvement of quality of life and prolongation of survival [4-6].

In the literature, there exists a large amount of information on radiotherapy during the disease course of metastatic BC. However, most of the published studies focused on the effect of different fractionation regimens and total radiation doses in the treatment of bone and brain metastasis, and spinal compression [4,7-9].

In a recent review, Budach evaluated the role of radiotherapy in the palliative BC situation in a more general approach and examined the various interventions in different anatomic sites [4]. This general approach, however, had never been applied to an institutional series of metastatic BC patients until now. To our knowledge, our study is the first to systematically evaluate how the available radiotherapy options were actually clinically implemented in an unselected cohort of BC patients with distant metastatic disease (DMD) over a longer period of time (1990-2012). By doing so, the main questions to be answered were: how often did patients receive BC-related radiotherapy in a situation considered to be incurable, at which metastatic sites, at what age, and in which phase of the disease course?

## Patients and methods

Data from the prospective relational Basel Breast Cancer Database (BBCD), which includes all newly diagnosed primary invasive BC cases treated at the University Women’s Hospital Basel, Switzerland since 1990, provided the basis for this study. This institution comprises the largest breast center in the canton of Basel and is representative of the population of the region. For this study, data from all female patients who were diagnosed with BC up to and including 2009 was analyzed (n = 1459).

During this 20-year period, 92 patients (6.3%) had DMD at initial diagnosis, or in other words, had primary metastatic disease (PMD). In 2011, with the exception of 37 patients who were lost to follow-up (2.5% of the entire study group), outcome information was available for all patients recorded in the BBCD. As of March 2011, 277 patients (20.3% of all patients who had stage I-III disease at initial BC diagnosis) had developed distant metastases over time, or in other words, had secondary metastatic disease (SMD). The median time between initial BC diagnosis and first diagnosis of DMD was 38.5 months (range: 2-215 months).

Out of 369 patients with confirmed distant metastatic BC, we were able to obtain information regarding the time of diagnosis of metastatic disease and date of death but we did not have complete information about the disease course and palliative therapy details for six patients (PMD, n = 1; SMD, n = 5). Thus, these patients were not considered for analysis, and ultimately 363 patients were included in the study (Table 1).

**Table 1:** Disease-related radiotherapy in breast cancer patients with distant metastatic disease

**Variable**	**n (%)**
Entire cohort of the BBCD	1459
Patients with distant metastases (DM)	369 (25.3)
PMD: DM at initial diagnosis	92 (6.3)
SMD: DM developed during the course of the illness	277 (19.0)
Patients with complete information about disease and therapy course.	363
Last follow-up: alive^1^	23 (6.3)
Study cohort: patients who died, i.e. patients with completed disease and therapy courses	340 (93.7)
	
Study cohort	340
A. Breast cancer-related radiotherapy	165 (48.5)
B. No radiotherapy	175 (51.5)
**Breast cancer-related radiotherapy**	
Number of patients	165
Number of series	255
median/mean (range)	1/1.6 (1-5)
Number of planning target volumes	337
median/mean (range)	2/2.0 (1-8)
**Metastatic sites and radiation volumes**	337
Breast and locoregional lymph nodes	35 (10.4)
- primary tumor region after surgery (PMD)	8 (2.4)
- primary breast tumor without surgery (PMD)	3 (0.9)
- recurrence at the chest wall, no surgery	6 (1.8)
- chest wall after surgery for local recurrence	1 (0.3)
- recurrence at locoregional lymph nodes	17 (5.0)
Bone	217 (64.4)
- vertebrae and osseous pelvis	137 (40.7)
- other sites	80 (23.7)
Brain	57 (16.9)
Other locations	28 (8.3)
- skin/soft tissue	13 (3.8)
- mediastinum	6 (1.8)
- eye	3 (0.9)
- lung	3 (0.9)
- cervical lymph nodes	2 (0.6)
- liver	1 (0.3)

BBCD: Basel Breast Cancer Database; DM: distant metastases; PMD: primary metastatic disease; SMD: secondary metastatic disease.

^1^Last follow-up: January 2013. Three patients were alive with no evidence of disease. The long-time survival after pathologically confirmed distant metastatic disease of these three patients was as follows: i) 204 months after diagnosis of bone metastases, ii) 209 months after diagnosis of bone and lung metastases, iii) 230 months after diagnosis of lung metastases. Detailed description of these three cases in: [10].

The patients in this cohort were followed until death. Patients who remained alive were followed until January 2013, thus all surviving patients had a follow-up time of at least 24 months. The outcome status of the cohort (n = 363) was as follows: 1) died of metastatic BC: 316 patients (87.1%); 2) died of other causes: 24 patients (6.6%); 3) alive with metastatic disease: 20 patients (5.5%); and 4) alive, no evidence of disease: 3 patients (0.8%, Table 1).

In order to analyze radiation oncology procedures during the palliative therapy course, we examined only the 340 patients who ultimately died of their metastatic disease (PMD, n = 78; SMD, n = 262). In other words, we analyzed only completed disease and treatment courses (Table 1). The clinicopathologic features of these 340 patients are listed in Table 2.

**Table 2:** Clinicopathologic and outcome characteristics between a cohort of 340 breast cancer patients with distant metastatic disease and completed disease courses; A. patients who received breast cancer-related radiotherapy during palliative situation, B. no radiotherapy

**Variable**	**Group A**	**Group B**
	**Radiotherapy**	**No radiotherapy**
	**n = 165 (%)**	**n = 175 (%)**
**AJCC/UICC TNM stage at initial diagnosis**		
Stage I	17 (10.3)	16 (9.1)
Stage II	56 (34.0)	60 (34.3)
Stage III	54 (32.7)	59 (33.7)
Stage IV	38 (23.0)	40 (22.9)
**Histologic subtype**^**1**^		
Ductal invasive	130 (79.3)	133 (76.9)
Lobular invasive	30 (18.3)	32 (18.5)
Rare types	4 (2.4)	8 (4.6)
Not available	1	2
**Grading**^**1**^		
G1/2	54 (34.6)	57 (35.2)
G3	102 (65.4)	105 (64.8)
Not available	9	13
**Hormonal receptor status**^**1**^		
Positive	116 (74.8)	125 (74.4)
Negative	39 (25.2)	43 (25.6)
Not available	10	7
**HER2 status, 2002-2009**^**1,2**^	**n = 40 (%)**	**n = 57 (%)**
Positive	16 (40.0)	10 (17.9)
“Triple-negative” carcinoma	6 (15.0)	12 (21.4)
Not available	-	1
**Metastatic disease survival**		
<12 months	80 (45.7)	46 (27.9)
12-24 months	33 (18.9)	34 (20.6)
25-48 months	43 (24.5)	58 (35.1)
>48 months	19 (10.9)	27 (16.4)

AJCC: American Joint Committee on Cancer; UICC: International Union Against Cancer [11,12].

^1^Histologic subtype, grading, hormonal receptor status and HER2 status were measured in primary breast tumor.

^2^Because HER-2 status has been routinely assessed for all patients since 2002, we only report data from 2002-2009 for this particular characteristic.

### Radiotherapy: definition of series and planning target volumes

For each case, the number of radiotherapeutic interventions (series) and the respective planning target volumes (ptv) were recorded. For example, a patient received radiotherapy for bone metastases in June 2005. In this first treatment series, two ptv were irradiated: the humerus with 15 Gy and a section of the thoracic and lumbar spine (T4-L1) with 30 Gy. In a second series in January 2006, the brain (30 Gy) and two further bone volumes- right femur (24 Gy) and a segment of the cervical and thoracic spine (C3-T1, 30 Gy)- were irradiated. In this particular case, we recorded two radiotherapy series and five ptv.

### Ethics committtee

The study design and data collection methods were approved by our institutional review board (*Ethikkommission beider Basel*).

### Statistical analysis

Since the ages of all subsets were found to have almost a Gaussian distribution, statistical differences between age of the subsets were analyzed using the unpaired t-test. The radiotherapeutic approaches and the survival times after radiotherapeutic interventions were compared by means of the nonparametric Wilcoxon-Test. Comparisons between nominal parameters were made with the Fisher exact test. In all statistical tests the level of significance was p < 0.05. Statistical evaluations were performed with Splus software (Version 6.1, Insightful Corporation, Seattle, WA, USA).

## Results

From a study cohort of 340 patients, 165 received radiotherapy as a part of palliative care. (48.5% of all patients with distant metastases and completed therapy and disease courses; Table 1).

### Radiotherapy vs. no radiotherapy

The patients who received radiotherapy during the palliative situation were significantly younger compared to those who had had no radiation (median age: 61 years [range: 30-89] vs. 68 years [range: 28-94], p < 0.001).

Patients who had received palliative radiation had a significantly improved metastatic disease survival (MDS; median: 26 months [range: 1-126] vs. 14 months [range: 0.5-102], p < 0.001). When one compares both groups with regard to a MDS of ≥24 months, a significantly higher percentage of patients who had radiation during the palliative disease course reached this mark compared to patients who had no radiation (52.1% vs. 37.1%, p = 0.006).

### Radiotherapy within palliative therapy and disease course of metastatic BC

Among the 165 patients who received radiotherapy, a total of 255 series with 337 ptv were applied (Table 1). The most common sites for radiotherapy were the bone (217 volumes, 64.4% of all radiated volumes) and the brain (57 volumes, 16.9%). Thirty-five ptv (10.4%) were applied in different locations with regard to the breast/chest wall, including regional lymph nodes. A further 28 volumes (8.3%) were applied to other locations (skin/soft tissue, mediastinum, eye, lung, cervical lymph nodes, liver; Table 1).

Table 3 shows the distribution of the metastatic sites. Bone metastases, which were diagnosed in nearly two thirds of the cases (69.6%), were the most frequent metastatic location, followed by metastases of the lung (51.5%), liver (43.8%), lymph nodes (28.5%) and brain (18.8%). With regard to the occurrence of metastatic lesions, brain (85.9%) and bone metastases (45.6%) were the most frequent radiated locations (Table 3). When metastases of the lung and the liver were diagnosed, they were treated with radiotherapy comparatively rarely (≤1%).

**Table 3:** Metastatic sites and radiotherapy

**Metastatic sites**	**Number of patients**	**Number of patients who had radiotherapy**
	**(% of the study cohort, n = 340)**	
		**(% of the metastatic site occurrence)**
Bone	237 (69.7)	108 (45.6)
Lung	175 (51.5)	1 (0.6)
Liver	149 (43.8)	1 (0.7)
Brain	64 (18.8)	55 (85.9)
Lymph nodes (excluding	97 (28.5)	8 (8.2)
ipsilateral locoregional LNs)		
Other locations	60 (17.6)	9 (15.0)
Local recurrence (breast and/or ipsilateral locoregional LNs)^1^	66 (19.4)	22 (33.3)

LN: lymph node.

^1^Only cases with local recurrences which were diagnosed and had radiotherapy after the diagnosis of other distant metastases.

Patients who had radiotherapy during their palliative disease course had a median age at the time of procedure of 60 years (mean age: 60.6 years, range: 32-89 years). There were no significant differences between the entire study group and patients who had radiotherapy for bone metastases (mean age: 60.5 years, p = 0.841); compared to the entire study cohort, patients who had radiotherapy for brain metastases were significantly younger (mean: 55.4 years, p = 0.011) (Table 4).

**Table 4:** Patient’s age at radiotherapy, time of radiotherapy within the disease course of metastatic breast cancer and survival after radiotherapy during the palliative situation

**Metastatic sites:**	**A. All cases**	**B. Bone**	**C. Brain**	**D. Local recurrence (incl. lymph nodes)**
	**255 series (%)**	**161 series (%)**	**57 series (%)**	
				**22 series (%)**
**Age (years)**				
Mean/median	60.6/60	60.5/58	55.4/57	62.6/64.5
(range)	(32-89)	(37-89)	(32-80)	(39-86)
**Phase of DMD**				
First third	127 (49.8)	88 (54.7)	16 (28.1)	15 (68.2)
Second third	44 (17.3)	26 (16.1)	15 (26.3)	2 (9.1)
Last third	84 (32.8)	47 (29.2)	26 (45.6)	5 (22.7)
**Series performed during:**				
Last 12 months of life	135 (52.9)	78 (48.4)	44 (77.2)	8 (36.4)
Last 6 months of life	95 (37.3)	53 (32.9)	33 (57.9)	5 (22.7)
Last month of life	29 (11.4)	15 (9.3)	10 (17.5)	2 (9.1)
**Survival after radiotherapy (months)**				
Mean/median	17.8/10	18.9/14	7.6/5	23.2/21
(range)	(0.2-123)	(0.2-121)	(0.2-29)	(0.5-99)
**Survival, p-values:**				
A vs. B: 0.318	B vs. C: <0.001			
A vs. C: <0.001	B vs. D: 0.346			
A vs. D: 0.158	C vs. D: <0.001			

DMD: distant metastatic disease; MDS: metastatic disease survival.

One hundred and twenty-seven out of 255 radiotherapy series (49.8%) were performed in the first third of the survival period (i.e. period of MDS); approximately one third of the procedures were performed in the last third (n = 84; Table 4). The median survival after radiotherapy was 10 months (range: 0.2-123 months) (Table 4). Patients who were radiated for bone metastases had a significantly longer survival time after the radiotherapeutic intervention compared to patients who had radiation for brain metastases (median: 14 months vs. 5 months, p < 0.001, Table 4).

Palliative systemic therapy: While patients who had radiotherapy during the palliative situation usually initiated systemic therapy (92.2%), patients who had no radiotherapeutic interventions received systemic therapy after the diagnosis of DMD significantly less often (81.7%, p = 0.006; Table 5). With regard to endocrine therapy, the number of patients who had radiotherapy and those who had not was comparable (radiotherapy: 61.7% vs. no radiotherapy: 57.7%, p = 0.149). Patients who did not have radiotherapy received palliative chemotherapy less often (73.3% vs. 53.7%, p < 0.001). In cases where palliative systemic therapy was applied, the median number of therapy lines was higher in the group of patients who received radiotherapy (3 vs. 2, p < 0.001; Table 5).

**Table 5:** Palliative systemic therapy and radiotherapy

	**No radiotherapy (175 patients)**	**Radiotherapy: all cases (165 patients)**	**Radiotherapy: bone (108 patients)**	**Radiotherapy: brain (55 patients)**
No systemic therapy	32 (18.3)	13 (7.8)	9 (8.3)	6 (10.9)
Chemotherapy (CT) only	42 (24.0)	44 (26.7)	24 (22.2)	26 (47.3)
Endocrine therapy (ET) only	49 (28.0)	31 (18.8)	23 (21.3)	3 (5.4)
CT + ET	52 (29.7)	77 (46.7)	52 (48.2)	20 (36.4)
Median number of systemic therapy lines	2 (1-8)	3 (1-10)	3 (1-10)	3 (1-10)

CT: chemotherapy; ET: endocrine therapy.

## Discussion

Most of the published studies on radiotherapy in metastatic BC evaluate only therapy options in pre-selected groups of patients with particular metastatic sites and focused on the effect of different fractionation regimens and total radiation doses [4]. In general, there are two different groups of palliative local radiotherapy, namely locoregional therapy of the primary tumor site and/or regional lymph nodes with or without surgery, and radiotherapy at distant anatomic sites:

1. Radiotherapy of the primary tumor site and/or regional lymph nodes with or without surgery

Traditionally, locoregional therapy in metastatic BC was limited to palliative management of uncontrolled disease such as large and ulcerative lesions. However, several retrospective studies indicated that patients who have primary DMD might benefit from the resection of the primary breast lesion in terms of prolonging survival [13-25]. These studies did not systematically consider the role of radiation in improving local control as a therapy concept; some authors did not even report on the rate of radiotherapy in their study cohorts [13,16,19,21-23], and the ones who did reported radiotherapy rates between 0%-67% [14,15,17,18,20,24,25]. Le Scodan et al. reviewed the clinical outcome of 581 patients with metastatic BC and focused more on the role of radiotherapy. The authors found that locoregional therapy, mainly consisting of radiotherapy, was also associated with a substantial gain in median survival [26]. In this retrospective analysis, 320 patients received locoregional treatment after diagnosis of DM: the majority of the patients (n = 249, 78%) received exclusively radiotherapy, 41 patients (13%) had radiotherapy of the breast or the chest wall after surgical removal of the tumor, and 30 patients (9%) had surgery alone. The authors found a survival benefit in favor of locoregional treatment of 11 months. The most favorable outcome data (median survival time: 39 months; three-year overall survival rate: 52.6%) was achieved by the combined locoregional approach of surgery followed by radiotherapy [26].

Le Scodan and colleagues used a multivariable analysis which controlled for a multitude of possible confounding factors such as age, clinical nodal status, metastatic patterns and use of systemic therapy. However, the hypothesis that improved locoregional tumor control may result in a survival benefit remains controversial because the results of retrospective data might be biased by one crucial confounder, namely the physicians’ selection for or against locoregional therapy. To date, no data from randomized trials are available to provide a higher level of evidence regarding the impact of locoregional therapy on survival in BC patients with primary DMD [4,27] and thus the decision whether or not to integrate breast surgery and/or radiotherapy of the primary tumor site into the palliative therapy concept needs to be done on an individual basis [4].

In our study cohort, the primary tumor was surgically removed at the time of diagnosis in 43 of the 92 patients (46.7%) who had primary DMD. From these 43 patients, eight (18.6%) received postoperative radiation of the breast/chest wall and or locoregional lymph nodes. In three patients, the primary breast lesion was not surgically removed but was later irradiated after tumor progression under systemic therapy (duration of systemic palliative therapy: six months in two patients, twelve months in one patient).

2. Radiotherapy at various distant sites

In contrast to locoregional therapy at the primary tumor site, which is mainly performed when DMD is first diagnosed, radiation of distant metastatic sites is usually performed at a later time point in the course of DMD. These cases comprise a highly heterogeneous group of radiotherapeutic interventions. Most studies on this subject evaluated the feasibility of different radiation schedules and outcome data of radiotherapy at the respective metastatic sites [4,7-9], classically the bone and brain, and for spinal cord compression. In doing so, these studies primarily reflect the perspective of one oncological subdiscipline, namely radiation oncology. However, they did not utilize control groups of patients with metastases at the same site who were not radiated, nor take into account the overall course of DMD. Thus, they failed to answer basic questions such as “How many BC patients with bone or brain metastases can be expected to have radiotherapy during their palliative disease course?” or “How are these procedures embedded in the entire disease and therapy course?”. These questions require a general oncologic perspective and can only be answered through examination of a cohort of unselected patients with metastatic disease and by thorough analysis of metastatic patterns.

In this study, we applied such a general oncologic approach. Based on a prospective BC database in which most of the patients who developed DMD were actually recorded (lost to follow-up rate of <3%) and in which the vast majority of palliative courses were completely documented with regard to metastatic patterns, systemic therapy, surgery and radiotherapy, we aimed to give a comprehensive overview regarding the totality of all disease-related radiotherapeutic procedures in the palliative BC situation.

In our study cohort comprised of 340 patients with distant metastatic BC, 48.5% of the patients had radiation oncology procedures during the palliative disease course. Approximately 50% of the procedures were performed in the first third of the palliative disease course and the median survival after radiation therapy was 10 months.

When interpreting our results, the following limitations of our study must be considered. First, our study comes from a single region of a small country with a high socioeconomic status. Secondly, our study analyzes retrospective data. On the other hand, it is a particular strength of our study that, besides the above mentioned valuable feature of complete documentation of disease course, we included patients who are usually underrepresented in large BC databases and thus are underreported in the oncologic literature, namely those who did not have any treatment from specialized oncologists, and did not receive surgery, radiotherapy and/or antineoplastic therapy.

The actual clinical use of radiotherapy in BC is dictated by both the surgeons’ and oncologists’ knowledge of indications for radiotherapy which determines the referral practice to a radiooncological therapy unit and the therapy principles of the respective unit. The data on radiotherapy in metastatic BC reported in this study might reflect a certain attitude towards palliative radiotherapy at our institution. In the palliative BC setting, there is currently no standard of care for this heterogeneous group of patients, and treatment decisions are made on an individual basis. In this scenario, it is easy to imagine that particular regional or even site-specific attitudes towards palliative therapy options might influence therapy decisions considerably more than in the adjuvant situation with its more clearly defined and widely accepted therapy guidelines. Thus, the rates of radiotherapeutic procedures reported in this study might vary from those of other cohorts of metastatic BC patients treated elsewhere.

In cases of metastatic cancer in which palliative therapy results in longer survival times, some essential aspects in the disease course and therapy concept resemble those of chronic non-malignant diseases. Chronic diseases are by definition long-lasting or recurrent and require a long period of treatment, supervision, observation or care; they are caused by non-reversible pathological alterations, leave residual disability, and can be altered but not be cured by various therapies [28,29]. Both chronic non-malignant diseases and longer metastatic disease courses require periodic therapy to control progressive course, and symptoms can be treated using strategies that permit stabilization with treatment regimens that have limited cumulative toxicity. There is no generally accepted definition as to how long a disease must last in order to be considered as chronic. In the case of rapidly progressive malignant diseases which lead to death within a few months, this is surely not justified. Undoubtedly, through the introduction of a new generation of effective agents with safer profiles in the last 20 years (e.g., endocrine therapy: third-generation aromatase inhibitors, fulvestrant; chemotherapy: taxanes, capecitabine, liposomal doxorubicin, gemcitabine, vinorelbine; immunotherapy: trastuzumab) and of course, through considerable advances in supportive care, longer survival times could be achieved, which in turn allows application of chronic disease treatment concepts in metastatic BC. In this study, which spanned an observation period of more than 20 years, the median MDS was 19 months. Of the 340 metastatic BC patients who ultimately died of the disease, 151 patients (44.4%) lived for 24 months or longer after diagnosis of DMD. One cannot assess exactly the impact of radiotherapy on increased survival rates in metastatic BC. We have deliberately foregone drawing conclusions regarding the impact of palliative surgery on survival. In addition to the retrospective approach of our study, there is a high degree of heterogeneity within the entire cohort and the described particular subgroups, which would make any analysis regarding palliative radiotherapy and prognostic impact more than debatable. However, compared to rapidly progressive disease courses with short survival times, in cases where a longer MDS was achieved, radiotherapy was significantly more often a part of the multimodal palliative therapy than not: a) in cases with a MDS ≥24 months, 57% of the patients had radiotherapy during the palliative disease course; b) in the study subgroup of patients who had radiotherapy in the palliative situation, the number of patients who had a MDS ≥24 months was significantly higher compared to those who did not receive radiation (52.1% vs. 37.1%).

## Conclusions

In a cohort of BC patients who had primary or who developed secondary DMD, nearly one half of the patients received disease-related radiotherapy during the palliative disease course. In the last decade, metastatic cancer has become increasingly viewed as a chronic disease process. In a general palliative therapy approach, which allows for treatment of patients according to the principles of a chronic disease, radiotherapy has a clearly established role in the therapy concept.

## Competing interest

The authors declare that there are no financial or personal relationships with other people or organizations that could inappropriately influence the work reported or the conclusions, implications, or opinions stated.

## Authors’ contributions

All authors have made substantial contributions to conception and design of the study (KS, MG, DH, SE, UG), acquisition of data (UG), or analysis and interpretation of data (KS, MG, DH, SE, UG). They have been involved in drafting the manuscript or revising it critically for important intellectual content (KS, MG, DH, SE, UG) and have given final approval of the version to be published (KS, MG, DH, SE, UG). Each author have participated sufficiently in the work to take public responsibility for appropriate portions of the content. All authors read and approved the final manuscript.
